# Development of a Double-Antibody Sandwich ELISA for the Detection of HPV16 E6 Protein

**DOI:** 10.3390/diagnostics16132002

**Published:** 2026-06-26

**Authors:** Peiyang Ding, Mingyang Yan, Xue Wang, Haili Wang, Wenying Yan, Yanwei Wang, Jingming Zhou, Aiping Wang

**Affiliations:** 1 Longhu Laboratory of Advanced Immunology, Zhengzhou 450046, China; 2 School of Life Sciences, Zhengzhou University, Zhengzhou 450001, China; 3 Henan Key Laboratory of Immunobiology, Zhengzhou 450001, China

**Keywords:** human papillomavirus type 16, E6 protein, monoclonal antibody, hybridoma technology, double-antibody sandwich ELISA

## Abstract

**Background:** The HPV16 E6 oncoprotein facilitates the ubiquitin-mediated degradation of the tumor suppressor p53, constituting a pivotal mechanism underlying viral immune evasion, cellular immortalization, and ultimately, malignant transformation. This study aimed to develop a reliable detection tool for the HPV16 E6 protein. **Methods:** Recombinant GST-E6 and E6-His proteins were expressed and purified using a prokaryotic expression system. Female BALB/c mice were immunized with GST-E6, and two hybridoma cell lines (G11A11 and A4) stably secreting anti-HPV16 E6 monoclonal antibodies were generated via hybridoma technology. Antibody pairing experiments identified A4 and G11A11 as suitable for sandwich ELISA. The optimal detection system was established using A4 antibody at 2 μg/mL for coating, HPV16 E6-His at 5 μg/mL as the detection antigen, and G11A11-HRP at a 1:200 dilution as the detection antibody. To validate the reliability, Hacat-HPV16E6 cell lysates were tested in parallel with a commercial ELISA kit. **Results:** After purification, the titers of both antibodies reached 1:204,800. The lower limit of quantification (LOQ) was 4.79 ng/mL and the limit of detection (LOD) was 3.39 ng/mL. The comparison with the commercial kit showed good consistency, with percentage differences ranging from 20% to 40%, confirming that the established ELISA is reliable for quantitative detection. **Conclusions:** This study successfully yielded high-titer and highly specific anti-HPV16 E6 monoclonal antibodies and developed a specific double-antibody sandwich ELISA, thereby furnishing a technical foundation for both fundamental research and laboratory-based applications related to HPV16-associated tumors.

## 1. Introduction

Human papillomavirus type 16 (HPV16) is a paradigmatic high-risk HPV strain and a primary etiological agent in several malignancies, including cervical cancer and head and neck squamous cell carcinoma [1,2]. Central to its oncogenicity is the HPV16 E6 oncoprotein, which facilitates ubiquitination and proteasomal degradation of the tumor suppressor p53 [3]. This process impairs essential cellular functions such as apoptosis and DNA repair [4], thereby promoting malignant transformation. Given that E6 is constitutively expressed throughout the viral life cycle and during carcinogenesis [5], the development of a specific and highly sensitive detection method for this oncoprotein is crucial. Such a method would significantly advance research into HPV16 pathogenesis, enable monitoring of viral infection [6], and assist in assessing tumor progression [7] and prognosis [8].

The HPV16 E6 protein is constitutively expressed in tumor cells and serves as a direct indicator of viral oncogenic activity. In patient-derived cell lysates or tumor tissue homogenates, E6 protein concentrations typically fall within the ng/mL to μg/mL range, making detection feasible using conventional ELISA platforms. Unlike viral DNA testing, which merely confirms the presence of the virus, direct quantification of the E6 protein more accurately reflects the functional transformation state of the virus. Therefore, establishing an ELISA method suitable for detecting HPV16 E6 protein in cell lysates is of great value for studying viral oncogenic mechanisms and assessing E6 expression at the cellular level.

Current HPV diagnostics predominantly target viral DNA [9], with limited approaches available for the direct detection of the E6 oncoprotein [10]. Immunoassays, such as enzyme-linked immunosorbent assays (ELISAs), offer notable benefits including operational simplicity, high throughput, and cost-effectiveness, making them well-suited for large-scale clinical screening. However, the development of a highly sensitive and specific ELISA hinges on the availability of high-affinity and specific antibodies.

Monoclonal antibodies (mAbs) represent ideal reagents for immunodetection due to their homogeneity, specificity, and scalable production. In this study, we aim to express and purify recombinant HPV16 E6 protein using a prokaryotic system, generate anti-HPV16 E6 mAbs via hybridoma technology, and identify paired antibodies suitable for sandwich immunoassays. Our ultimate goal is to establish a robust and reliable double-antibody sandwich ELISA to support basic studies of HPV16 E6 protein [11,12].

## 2. Materials and Methods

### 2.1. Materials and Reagents

The plasmids pGEX-6P-1 and pet-28a(+) were maintained in our laboratory. Taq DNA polymerase, restriction enzymes, T4 DNA ligase, and associated reaction buffers were obtained from TaKaRa (San Jose, CA, USA). RPMI Medium 1640, fetal bovine serum, Hypoxanthine-Aminopterin-Thymidine (HAT) and Hypoxanthine-Thymidine (HT) selective supplements were sourced from Gibco (Waltham, MA, USA); 50% polyethylene glycol (PEG) solution was procured from Roche (Basel, Switzerland). Isopropyl β-D-1-thiogalactopyranoside (IPTG), imidazole, caprylic acid, FITC-conjugated goat anti-mouse IgG, HRP-conjugated goat anti-mouse IgG, and other routine chemical reagents were purchased from Sigma-Aldrich (St. Louis, MO, USA). A Protein A affinity chromatography column, Ni Sepharose™ Fast Flow, and Glutathione S-Transferase (GST) purification resin were acquired from GE Healthcare (Chicago, IL, USA); DMEM cell culture medium was obtained from Invitrogen (Carlsbad, CA, USA). Carbonate Buffer solution (CBS), phosphate-buffered saline (PBS), phosphate-buffered saline with Tween^®^ 20 (PBST), and blocking buffer (5% skim milk in PBST) were prepared in-house. BL21(DE3) and DH5α competent cells, as well as the 293T cell line, were provided by the Longhu Modern Immunology Laboratory (Zheng-zhou, China). In addition, a Hacat-HPV16E6 cell line stably transfected with the HPV16 E6 gene was established and maintained in our laboratory. The Human Papillomavirus Type 16 E6 Protein (HPV16 E6 protein) ELISA Kit (Catalog No. CB13752-Hu) was purchased from Keaibo Biological (Shanghai, China). According to the manufacturer’s instructions, this kit is designed for the quantitative detection of HPV16 E6 protein in serum, plasma, and cell culture supernatants, with a detection range of 0.312–10 ng/mL and a lower limit of quantitation (LLOQ) of <0.1 ng/mL. The kit employs a double-antibody sandwich format with proprietary antibodies and the HRP/TMB colorimetric detection system. All animal experiments were performed using female BALB/c mice obtained from the Laboratory Animal Center of Zhengzhou University (specific pathogen-free grade). The mice were experimentally naive prior to this study, and no genetic modifications were applied. For immunization, two female BALB/c mice (6–8 weeks old, weighing approximately 18–22 g) were used. All animal procedures were performed at the Laboratory Animal Facility of Longhu Laboratory, Zhengzhou, China.

### 2.2. Prokaryotic Expression and Purification of HPV16 E6 Protein

The HPV16 E6 gene fragment was subcloned into the pGEX-6P-1 and pET-28a(+) vectors to generate GST-E6 and E6-His recombinant plasmids, respectively. Following sequence verification, the plasmids were transformed into DH5α competent cells. After culture expansion and plasmid extraction, the constructs were introduced into BL21(DE3) competent cells for large-scale cultivation. Protein expression was induced by the addition of isopropyl β-D-1-thiogalactopyranoside (IPTG). Subsequently, the cells were harvested and lysed. The GST-E6 fusion protein was purified using a GST affinity column, while the E6-His protein was isolated employing Ni-NTA resin. The purified proteins were then characterized by sodium dodecyl sulfate–polyacrylamide gel electrophoresis (SDS-PAGE) and Western blot analysis. To validate the expression of HPV16 E6 transcript in the established stable cell line, total RNA was extracted from both HaCaT-HPV16E6 cells and negative control HaCaT cells, followed by reverse transcription to synthesize complementary DNA (cDNA). Quantitative real-time PCR (qPCR) was subsequently performed using the following primer pair: forward 5′-GCATCAGAAACGGACAGCCA-3′ and reverse 5′-TCAGGCACTTGTCGCAAACA-3′.

### 2.3. Generation and Purification of Monoclonal Antibodies

Purified GST-E6 protein was used as the immunogen. Two female BALB/c mice were immunized via subcutaneous multi-point injection with a 40 μg dose of protein emulsified in an equal volume of adjuvant; this process was repeated for a total of four immunizations. The initial immunization utilized Freund’s complete adjuvant, while subsequent booster immunizations employed Freund’s incomplete adjuvant. A final intraperitoneal booster immunization (without adjuvant) was administered. Tail vein blood was collected seven days after each immunization to monitor serum antibody titers. Three days after the final boost, mouse splenocytes were fused with SP2/0 myeloma cells. Hybridoma screening was performed using an indirect enzyme-linked immunosorbent assay (icELISA) with E6-His as the detection antigen to avoid anti-GST antibody interference. Positive hybridomas were subcloned using the limiting dilution method. Selected hybridoma cell lines stably secreting specific antibodies were then injected intraperitoneally into mice pre-treated with liquid paraffin to induce ascites production. For this purpose, two multiparous female BALB/c mice (approximately 7–9 months old) were pretreated with 0.5 mL of liquid paraffin via intraperitoneal injection 7 days prior to hybridoma cell inoculation. Each mouse received 1 × 10^6^ hybridoma cells (one mouse for the G11A11 cell line and the other for the A4 cell line) suspended in 0.5 mL of sterile phosphate-buffered saline (PBS) via intraperitoneal injection. After cell injection, the mice were monitored daily for signs of abdominal distension, pain, or distress. When ascites became evident (typically 7–10 days after cell injection), the ascitic fluid was collected using a sterile syringe. Monoclonal antibodies were subsequently purified from the ascitic fluid using a Protein A affinity chromatography column. A total of four female BALB/c mice were used in this study: two for immunization and two for ascites production.

### 2.4. Construction of the Hacat-HPV16 E6 Stably Transfected Cell Line

The HPV16 E6 fragment was PCR-amplified using primers designed to incorporate a myc tag at the C-terminus, and then inserted into the shuttle plasmid pLenti-GIII-CMV-CBH-GFP-2A-Puro via double restriction enzyme digestion and T4 DNA ligase-mediated ligation. This shuttle plasmid, along with the packaging plasmids psPAX2 and pMD2.G, was co-transfected into 293T cells. At 24 h post-transfection, the culture medium was replaced with fresh medium, and cells were continuously cultured. Cell morphology and transfection efficiency were monitored by fluorescence microscopy at 48 and 72 h post-transfection. Viral supernatants were collected at 48 and 72 h, pooled, and filtered through a 0.45 μm filter. Hacat cells seeded in 6-well plates were then infected with the filtered viral supernatant. At 72 h post-infection, cells were transferred to T25 culture flasks and maintained in DMEM complete medium containing half the standard concentration of puromycin (0.3 μg/mL). The following day, the medium was replaced with DMEM complete medium containing the full concentration of puromycin (0.6 μg/mL). After three rounds of selection, a Hacat cell line stably overexpressing the HPV16 E6 protein was successfully established. It is important to note that the lentiviral construct contained the full-length HPV16 E6 cDNA without any intronic sequences; therefore, the transcribed mRNA cannot undergo alternative splicing to produce splice variants. Consequently, only full-length E6 protein is expressed in this cell line.

### 2.5. Identification of Monoclonal Antibodies

#### 2.5.1. Titer Determination

Seven days post-immunization, approximately 10 μL of whole blood was collected from the tail vein of each mouse and immediately diluted 100-fold (10 μL blood + 990 μL phosphate-buffered saline [PBS]) to prevent clotting and to obtain a suitable dilution for antibody titer determination by indirect ELISA. His-tagged E6 protein was diluted to 2 μg/mL in coating buffer (CBS), added to a 96-well plate (100 μL/well), and incubated overnight at 4 °C. After washing with PBST (PBS containing 0.05% Tween-20), the plates were blocked with 5% skim milk at 37 °C for 2 h. Following another wash, serially diluted mouse serum samples were added and incubated at 37 °C for 1 h. The plates were washed again, and horseradish peroxidase (HRP)-conjugated goat anti-mouse IgG secondary antibody was added, followed by incubation at 37 °C for 30 min. After a final wash, the reaction was developed, and the absorbance was measured using a microplate reader (Bio-Rad Laboratories, Inc., Hercules, CA, USA).

#### 2.5.2. Immunofluorescence Assay

The pcDNA3.1-HPV16 E6-His plasmid was transfected into 293T cells. After 24–48 h, the cells were fixed and permeabilized. The purified monoclonal antibody was applied as the primary antibody, followed by incubation with FITC-conjugated goat anti-mouse IgG. Specific fluorescence signals were visualized and captured using a fluorescence microscope.

#### 2.5.3. Antibody Pairing Test

Selected monoclonal antibodies were conjugated with HRP. A checkerboard titration method was employed for pairing assessment: one unlabeled antibody was coated onto the ELISA plate, followed by the addition of HPV16 E6-His antigen and then a different HRP-conjugated antibody. The effectiveness of each antibody pair in forming a sandwich complex was evaluated based on the signal intensity of the 3,3′,5,5′-Tetramethylbenzidine (TMB) colorimetric reaction.

### 2.6. Establishment and Optimization of the Double-Antibody Sandwich ELISA

Based on the pairing results, the optimal antibody pair was selected to establish a double-antibody sandwich ELISA. The following parameters were systematically optimized.

#### 2.6.1. Determination of Optimal Antigen Detection Range

The selected capture antibody was diluted 1:200 in CBS, and 100 μL was added to each well of a 96-well plate in triplicate. After overnight coating at 4 °C and blocking with 5% skim milk, HPV16 E6-His protein was added at concentrations of 0, 1, 5, and 10 μg/mL (diluted in PBS). Finally, 100 μL of the corresponding HRP-conjugated detection antibody was added to each well, and the assay was developed.

#### 2.6.2. Optimization of Antibody Concentrations

A checkerboard titration was performed to determine the optimal working concentration for the coating antibody (A4: 1, 2, 4, 8 μg/mL) and the optimal dilution for the HRP-conjugated detection antibody (G11A11: 1:200, 1:400, 1:800, 1:1600). The capture antibody was coated at the specified concentrations. HPV16 E6 protein was diluted to its predetermined optimal concentration, and 100 μL was added per well. After incubation, 100 μL of the HRP-conjugated detection antibody at the corresponding dilution was added, and the absorbance was measured.

#### 2.6.3. Optimization of Coating Conditions

Four different coating conditions were evaluated: 37 °C for 2 h, 37 °C for 4 h, 4 °C for 2 h, and 4 °C overnight, each performed in triplicate. The condition yielding the highest positive-to-negative (P/N) ratio was selected as the optimal coating protocol.

#### 2.6.4. Establishment of Standard Curve and Determination of Sensitivity

The recombinant HPV16 E6-His protein was serially diluted in PBS to obtain the following concentrations: 187.5, 93.75, 46.875, 23.4375, 11.71875, 5.859375, 2.9296875, and 1.46484375ng/mL. Each concentration was tested in triplicate. The OD450 nm values were measured using a microplate reader, and a standard curve was generated. Blank control wells (containing PBS only, without antigen) were also included. The double-antibody sandwich ELISA was performed under the optimized reaction conditions described above, and the OD450 nm values were recorded. The limit of detection (LOD) was calculated as the mean OD value of the blank controls plus three times the standard deviation (SD), and the limit of quantification (LOQ) was defined as the mean OD value plus ten times the SD.

#### 2.6.5. Comparative Validation of the In-House ELISA and a Commercial Kit

Stable cells were collected and lysed in RIPA buffer on ice for 10 min. The lysates were centrifuged at 12,000× *g* for 20 min at 4 °C, and the supernatants were divided into two aliquots: one was analyzed by the double-antibody sandwich ELISA established in this study, and the other was measured in parallel using a commercial kit (Keaibo Biological, Shanghai, China).

### 2.7. Data Processing

All experimental data, including serum antibody titers, ascites collection, ELISA optimization (OD450 values, P/N ratios, standard curve fitting), and cell lysate validation data, were processed using Microsoft Excel 2019 (Microsoft Corporation, Redmond, WA, USA) and GraphPad Prism 8.0 (GraphPad Software, San Diego, CA, USA). Endpoint titers were determined as the highest serum dilution yielding an OD450 value greater than 2.1 times the mean OD of the negative control.

## 3. Results and Analysis

### 3.1. Establishment and Validation of HPV16 E6 Expression Systems

**Figure 1:**
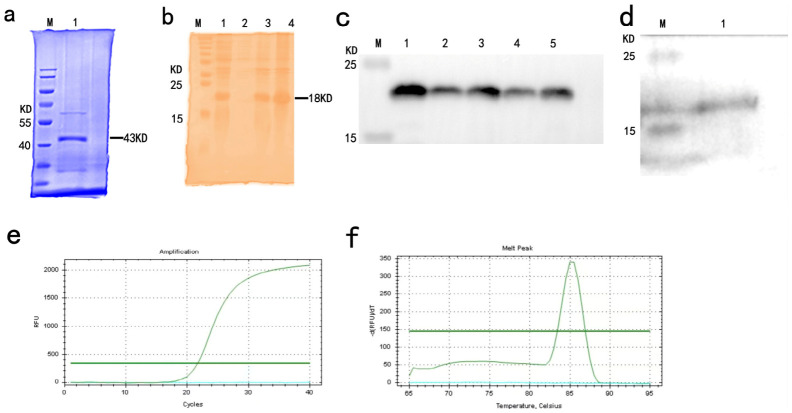
Expression and purification analysis of recombinant HPV16 E6 proteins, Western blot validation of E6 protein expression in the stable cell line, and qPCR validation of E6 transcript. (**a**) SDS-PAGE analysis of GST-HPV16 E6: Lane 1, soluble supernatant. (**b**) SDS-PAGE analysis of HPV16 E6-His: Lane 1, flow-through; Lane 2, wash fraction; Lane 3, elution fraction with 400 mM imidazole; Lane 4, elution fraction with 500 mM imidazole. Both (**a**) and (**b**) indicate predominant soluble expression. (**c**) Western blot analysis under reducing conditions, confirming the identity of the purified HPV16 E6-His protein from the soluble fraction (Lanes 1–5: representative elution fractions) using an anti-His tag antibody. (**d**) Western blot analysis of HaCaT-E6-Myc stable cell line lysates using an anti-Myc tag antibody: Lane 1, cell lysate sample showing the E6 protein (18 kDa) at the correct position. (**e**) Amplification curves of quantitative real-time PCR (qPCR). The x axis represents the cycle number, and the y axis represents the fluorescence intensity. One S-shaped curve (experimental sample) and one horizontal line (control sample) are shown in the plot. (**f**) Melting curves. The x-axis represents temperature (°C), and the y-axis represents the negative first derivative of fluorescence (dF/dT). The experimental sample shows a distinct characteristic peak, while the control sample appears as a flat line.

After purification of GST-HPV16 E6 and HPV16 E6-His recombinant proteins, SDS-PAGE and Western blot analyses were performed. The results showed that GST-HPV16 E6 (43 kDa) appeared at the correct position and was expressed in the supernatant (Figure 1a). For HPV16 E6-His (18 kDa), the SDS-PAGE result showed the expected band at the correct position (Figure 1b), and Western blot analysis further confirmed its expression in the supernatant (Figure 1c).

### 3.2. Characterization of Hacat-HPV16E6 Stable Cells

To confirm that the Hacat-HPV16E6 cell line expresses only full-length E6 protein and not splice variants, whole-cell lysates were subjected to Western blot analysis using an anti-Myc tag antibody (as shown in Figure 1d). A single band of approximately 18 kDa, corresponding to full-length HPV16 E6, was detected. No smaller bands that would indicate the presence of splice variants were observed. Furthermore, qPCR analysis was performed using total RNA extracted from HaCaT-HPV16E6 cells and negative control HaCaT cells. As shown in Figure 1e, the amplification curve of the experimental group exhibited a typical S shape after a certain number of cycles, whereas the negative control group remained flat, indicating successful amplification of the target fragment. In the melting curve analysis (Figure 1f), the experimental group displayed a single, specific peak with a melting temperature of approximately 85 °C, while the negative control showed no distinct peak. These results demonstrate that the cell line specifically expresses full-length E6 transcript. This result confirms that, due to the use of intronless E6 cDNA, the stable cell line exclusively produces full-length E6 protein, thereby eliminating potential interference from splice variants in subsequent ELISA validation.

### 3.3. Screening of Hybridoma Cell Lines and Preparation of Monoclonal Antibodies

**Figure 2:**
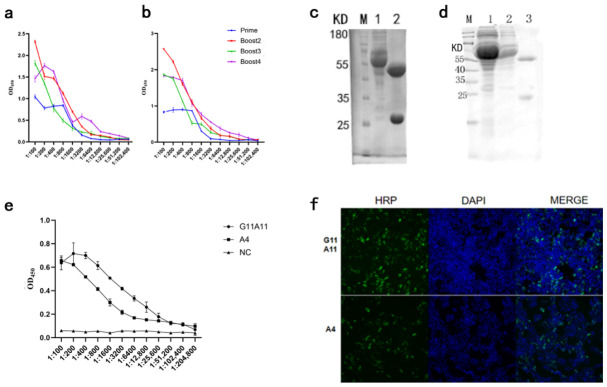
Assessment of Immune Responses and Monoclonal Antibody Characterization. (**a**) Antibody titers in mouse A after prime and booster immunizations. Serum dilution factor is plotted on the x-axis, and OD450 values are plotted on the y-axis. The lines in different colors represent sera from the prime immunization and the third booster immunization. (**b**) Antibody titers in mouse B after prime and booster immunizations, presented as in (**a**). Both mice achieved a maximum titer of 51,200. (**c**) SDS-PAGE analysis of the A4 monoclonal antibody purification process. Lane 1: flow-through; Lane 2: elution. (**d**) SDS-PAGE analysis of the G11A11 monoclonal antibody purification. Lane 1: flow-through; Lane 2: wash fraction; Lane 3: elution. (**e**) Titers of the purified A4 and G11A11 antibodies as determined by indirect ELISA. Both antibodies exhibit a titer of 204,800. (**f**) Immunofluorescence staining demonstrating the specific binding of A4 and G11A11 antibodies to eukaryotically expressed HPV16 E6 protein (green).

As shown in Figure 2a,b, the serum titers of Mouse A and Mouse B both reached 1:51,200 after four immunizations. After cell fusion and three rounds of subcloning, five hybridoma cell lines secreting high-titer HPV16 E6 monoclonal antibodies were finally obtained, named G11A11, A4, C2B2, C1, and C3. Two of them (G11A11, A4) were selected for ascites preparation and purification via Protein A columns. The results showed that high-purity antibodies were obtained (Figure 2c,d).

### 3.4. Characterization of Monoclonal Antibodies

#### 3.4.1. Antibody Titer and Immunofluorescence Analysis

The purified monoclonal antibodies G11A11 and A4 both exhibited high titers. As shown in Figure 2e, the OD values remained above the negative control at a dilution of 1:204,800, indicating that the titers reached at least 1:204,800. Detailed data are provided in Supplementary Table S1. In addition, specific green fluorescence was observed in 293T cells transfected with pcDNA3.1-HPV16 E6-His when stained with either G11A11 or A4 (Figure 2f), confirming that both antibodies can specifically recognize eukaryotic-expressed HPV16 E6 protein.

#### 3.4.2. Antibody Pairing

As shown in Table 1, when A4 was used as the coating antibody and G11A11-HRP as the detection antibody, the G11A11-A4 pair produced the strongest signal and the highest P/N value, indicating that these two antibodies recognize different epitopes of the HPV16 E6 protein and can form an effective sandwich pair.

### 3.5. Optimization of Double-Antibody Sandwich ELISA Conditions

#### 3.5.1. Determination of Optimal Antigen Concentration

**Figure 3:**
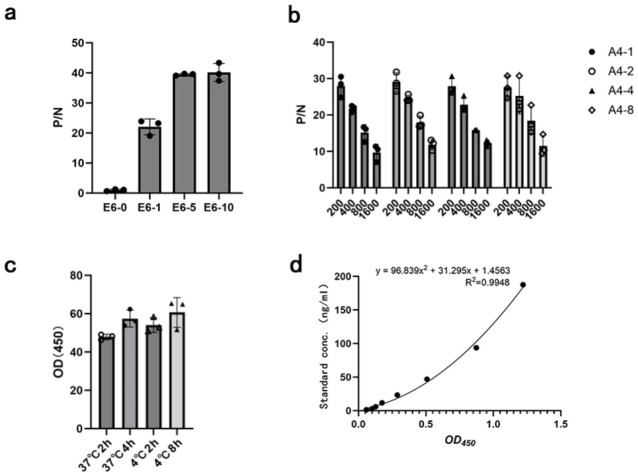
Sandwich ELISA Optimization. (**a**) Antigen titration. Titration of HPV16 E6 antigen determined 5 μg/mL to be the optimal concentration as it yielded the highest P/N ratio. (**b**) Antibody concentration. The assay sensitivity was maximized using a capture antibody (A4) concentration of 2 μg/mL and a detector antibody (G11A11-HRP) dilution of 1:200. (**c**) Coating condition. Among the conditions tested, coating at 4 °C overnight produced the highest absorbance and was subsequently used. (**d**) Standard curve and quantitative analysis. A standard curve was generated by plotting the OD value against the E6 concentration. Polynomial curve fitting yielded a high correlation coefficient (R^2^ = 0.9948).

A4 monoclonal antibody was used as the coating antibody in ELISA wells. Then, E6-His concentration was set at three gradients with one negative control group, each with three replicates. As shown in Figure 3a, when the E6-His concentration reached 5 μg/mL, the highest P/N value was achieved. Subsequent experiments used an E6-His concentration of 5 μg/mL. Importantly, the P/N value increased monotonically with antigen concentration up to 10 μg/mL without any decline, indicating the absence of a post-zone effect within this concentration range. Detailed data are provided in Supplementary Table S2.

#### 3.5.2. Optimal Enzyme-Labeled Antibody and Coating Antibody Concentrations for Double-Antibody Sandwich ELISA

As shown in Figure 3b, when the coating antibody A4 concentration was 2 μg/mL, the detection antigen E6-His concentration was 5 μg/mL, and the detection antibody G11A11-HRP dilution ratio was 1:200, the P/N value was the highest, indicating the optimal working conditions. Detailed data are provided in Supplementary Table S3.

#### 3.5.3. Coating Conditions

Four combinations of temperature and time were set for A4 antibody coating. The OD450 value for coating overnight at 4 °C was higher than that for coating at 37 °C for 2 h/4 h or at 4 °C for 2 h (Figure 3c). Therefore, the optimal coating condition was determined to be overnight at 4 °C.

#### 3.5.4. Standard Curve and Sensitivity Assay Results

E6-His standard solutions were prepared in gradients and detected by the double-antibody sandwich ELISA to establish a standard curve (mean of three replicates). As shown in Figure 3d, the standard curve fitting R^2^ reached 0.9948, indicating a good fit of the experimental data to the polynomial curve. Detailed data are provided in Supplementary Table S4. As shown in Table 2, the lower limit of quantification (LOQ) concentration was approximately 4.79 ng/mL, and the limit of detection (LOD) measured when the sample was PBS was approximately 3.39 ng/mL. It is important to clarify that this LOD value represents the minimum concentration of HPV16 E6 protein that can be reliably distinguished from the PBS buffer background under the established assay conditions.

#### 3.5.5. Validation of the Double-Antibody Sandwich ELISA for Detecting HPV16 E6 Protein Expression

The results showed that the OD values obtained from the 16-fold diluted samples measured by the commercial kit were comparable to those obtained from the 2-fold diluted samples measured by the established method, with percentage differences ranging from 20% to 40% (Table 3 and Table 4). These findings demonstrate that the established double-antibody sandwich ELISA is capable of reliably quantifying HPV16 E6 protein, making it suitable for detecting this target protein in biological samples.

To quantitatively compare the sensitivities of the two methods, we determined the dilution factors required to achieve comparable OD values. As shown in Table 3 and Table 4, the commercial kit exhibited approximately 8-fold higher analytical sensitivity than our developed ELISA (16-fold dilution vs. 2-fold dilution, respectively).

## 4. Discussion

This study successfully generated three specific monoclonal antibodies by immunizing mice with a prokaryotically expressed GST-HPV16 E6 fusion protein and applying hybridoma technology. During the screening process, the GST tag was removed from the HPV16 E6-His protein, which was then used as the detection antigen. This approach effectively prevented interference from anti-GST antibodies and ensured that the selected monoclonal antibodies specifically recognized the HPV16 E6 protein itself.

Among the three monoclonal antibodies obtained, G11A11 and A4 exhibited the highest titers (1:204,800) and demonstrated favorable pairing characteristics. Immunofluorescence assays confirmed that these antibodies not only recognized prokaryotically expressed E6 protein but also effectively bound to HPV16 E6 protein expressed in eukaryotic cells, indicating that their target epitopes are exposed in the native conformation. This property underscores their potential for use in detecting clinical samples, such as tissue sections [13,14].

In establishing the double-antibody sandwich ELISA, we systematically optimized key parameters including coating conditions and antibody concentrations [15], which significantly enhanced detection sensitivity while minimizing background noise. To evaluate the practical applicability of the established method, we further validated its performance using Hacat-HPV16 E6 cell lysates as test samples. The results demonstrated that the established ELISA reliably detected HPV16 E6 protein in stably transfected cells, with measurements showing good consistency compared to a commercial kit (percentage differences within 20–40%), confirming its suitability for detecting the target protein in biological samples. The direct detection of HPV16 E6 protein has garnered increasing attention, as it reflects viral oncogenic activity [16,17] and, compared to viral DNA testing, may more accurately indicate the state of malignant transformation [18]. Stable and highly specific monoclonal antibodies serve as core reagents for the development of such detection platforms [19].

In the current optimization, a stepwise strategy was used: antigen titration (Figure 3a) showed no signal decline up to 10 μg/mL, indicating no obvious prozone effect within this range. Based on this, the antigen concentration was fixed at 5 μg/mL for subsequent antibody titrations. A full three-dimensional titration was not performed, which represents a limitation of the current study. However, the antigen concentration range tested (0–10 μg/mL) covers the expected levels in our cell lysate model. Additionally, although our antigen titration experiment showed no post-zone effect up to 10 μg/mL, the possibility of a hook effect at higher antigen concentrations cannot be completely excluded. For future clinical applications, serial dilution of patient samples will be employed to detect and mitigate any potential hook effects.

We compared our ELISA with a commercial kit (Xinyu Bio) [20] and a fluorescence immunochromatography method (Sheng Wu Gong Cheng Xue Bao, 2024) [21]. As summarized in Table 5, our assay shows a detection limit of 3.39 ng/mL, which is higher than that of the commercial kit (<1.0 ng/mL) and the fluorescence method (1.09 ng/mL). This difference is primarily due to our use of a conventional HRP/TMB colorimetric system without signal amplification, whereas the fluorescence method employs time-resolved fluorescence detection. Nevertheless, our assay offers distinct advantages: it uses well-characterized in-house monoclonal antibodies (A4 and G11A11), is cost-effective, and requires no specialized equipment, making it suitable for routine laboratory use. Future optimization, such as signal amplification, is expected to enhance sensitivity.

As noted in the Results (Section 3.5.5), the commercial kit exhibited approximately 8-fold higher sensitivity than our ELISA. This difference can be attributed to process optimization (e.g., high protein-binding microplates, proprietary buffers) and rigorous manufacturing standardization of the commercial product, which enhance antigen capture efficiency and signal-to-noise ratios. Despite this, our assay offers the advantage of using well-characterized in-house monoclonal antibodies, providing a cost-effective and sustainable foundation for future optimization.

Current methods for detecting and typing high-risk HPV mainly include p16 immunohistochemistry [22], HPV DNA testing for the L1 gene, mRNA detection [23], and cytomorphological evaluation [24,25]. Although p16 detection is widely used to distinguish high-risk from low-risk HPV infections [26,27], it is not entirely specific—upregulation of p16 can be induced not only by HPV16 E7 but also by E7 proteins from other high-risk HPV types [28,29]. Similarly, L1-based DNA or mRNA testing does not directly reflect the presence or expression levels of the HPV16 E6 oncoprotein [30] and may carry a risk of false positives. In contrast, the double-antibody sandwich ELISA developed in this study enables direct detection and quantitative measurement of HPV16 E6 protein in cells [31]. As a direct marker of viral subtype and an indicator of infection stage [32], this assay offers superior specificity [33] and enhanced clinical relevance [34].

The current ELISA system was optimized using recombinant HPV16 E6 protein diluted in PBS and validated with HaCaT-HPV16E6 cell lysates. Therefore, the reported limit of detection (LOD = 3.39 ng/mL) and limit of quantification (LOQ = 4.79 ng/mL) are specifically applicable to samples prepared in PBS or similar simple buffers, such as cell lysates from cultured cells. Potential matrix effects may arise when the assay is applied to more complex biological samples [35] (e.g., tissue homogenates with high protein content). In such cases, routine practices including sample dilution, the use of blocker-containing diluents, or the addition of mild detergents (e.g., Triton X-100) could help mitigate interference. It should be emphasized that this assay is designed for laboratory-based detection of HPV16 E6 protein in cell lysates (e.g., from transfected or infected cell lines) and is not intended for detecting circulating E6 in patient serum or plasma, where E6 concentrations are typically in the picogram per milliliter range—far below the current LOD. Future work may explore adapting the antibody pair for other formats (such as immunohistochemistry) or incorporating signal amplification strategies to enhance sensitivity for specific research needs.

Regarding the validation using clinical specimens, we acknowledge that the current study has not yet included immunostaining data on HPV16-positive patient tissues, such as oropharyngeal cancer or cervical cancer samples. While our immunofluorescence assay in 293T cells transfected with HPV16 E6 (Figure 2f) demonstrates that both G11A11 and A4 antibodies recognize eukaryotic-expressed E6 protein, we acknowledge that testing on clinical specimens is essential to establish diagnostic applicability. Therefore, in our future work, we will collect HPV16-positive and HPV-negative tumor tissues to perform immunohistochemical staining using the monoclonal antibodies generated in this study.

The sensitivity, specificity, and predictive values of our ELISA have not been determined using a large clinical cohort (≥100 patients) due to lack of sample access. This is a major limitation of the current proof-of-concept study. Future clinical collaboration will be pursued to perform this essential validation. More importantly, the anti-HPV16 E6 monoclonal antibodies generated in this study have broader applications beyond ELISA. Immunofluorescence assays have already demonstrated their ability to recognize eukaryotic-expressed E6 protein, suggesting their potential utility in immunohistochemistry (IHC). We are actively collecting HPV16-positive and -negative clinical tumor tissue samples to perform IHC staining using the A4 and G11A11 antibodies in order to evaluate their diagnostic applicability in histology. These clinical validation studies will be reported in due course.

## 5. Conclusions

This study successfully generated a panel of high-titer and highly specific mouse-derived monoclonal antibodies against the HPV16 E6 protein, from which the G11A11 and A4 antibody pair was identified as suitable for sandwich immunoassays. Using this pair, a double-antibody sandwich ELISA was systematically optimized and preliminarily established. The development of this assay provides an essential material foundation and a practical technical platform for further investigation into the biological functions of HPV16 E6 and supports the future development of diagnostic kits for HPV16-related tumors.

## 6. Patent

A patent application covering the G11A11 monoclonal antibody reported in this manuscript is under preparation. No application has been filed as of the time of this submission.

## Figures and Tables

**Table 1:** Pairing analysis of anti-HPV16 E6 monoclonal antibodies. Cross-pairing assay was performed to identify antibody pairs recognizing distinct epitopes. The combination of A4 (capture) and HRP-conjugated G11A11 (detection) yielded the highest P/N value, indicating that these two antibodies recognize non-overlapping epitopes suitable for sandwich ELISA.

Ab ID	HRP-Ab		
	G11A11-HRP	A4-HRP	Neg Ctrl
G11A11	0.0759	0.271	0.0652
A4	0.3871	0.0931	0.051

**Table 2:** Determination of the limit of detection (LOD) and limit of quantitation (LOQ) for the double-antibody sandwich ELISA. Twelve negative control samples (PBS) were measured using the established double-antibody sandwich ELISA at 450 nm. The mean OD value and standard deviation (SD) were calculated. The LOD, defined as the mean plus 3 SDs, was determined to be 3.39. The LOQ, defined as the mean plus 10 SDs, was determined to be 4.79. These values represent the thresholds for specific detection and reliable quantitation of HPV16 E6 protein, respectively.

BLANK OD_450_			
0.0386	0.0428	0.0392	0.0441
0.0431	0.0448	0.0318	0.0347
0.0434	0.0421	0.0344	0.0352

**Table 3:** Detection of HPV16 E6 protein in Hacat-HPV16E6 cell lysates using a commercial ELISA kit. Samples were diluted 16-fold prior to assay. OD values were measured at 450 nm using a microplate reader. Concentrations were interpolated from the standard curve provided with the kit. The commercial kit has a lower limit of quantitation (LLOQ) of 0.3125 ng/mL.

Sample	Measured (16×)	Conc.	Original Conc.
1	0.048	0.682864	10.925824
2	0.0443	0.5028849	8.0461584
3	0.0415	0.3666845	5.866952

**Table 4:** Detection of HPV16 E6 protein in Hacat-HPV16E6 cell lysates using the established double-antibody sandwich ELISA. Samples were diluted 2-fold prior to assay. OD values were measured at 450 nm using a microplate reader. Concentrations were interpolated from the standard curve established in this study. The established method has a lower limit of quantitation (LLOQ) of 4.79 ng/mL.

Sample	Measured (2×)	Conc.	Original Conc.
1	0.1325	7.303017194	14.60603439
2	0.0938	5.243803131	10.48760626
3	0.0613	3.738574442	7.477148884

**Table 5:** Comparison of three methods for HPV16 E6 protein detection: in-house double-antibody sandwich ELISA, commercial ELISA kit, and fluorescence immunochromatography. The table summarizes the analytical performance of the method developed in this study versus two other representative methods (a commercial ELISA kit and a fluorescence immunochromatography assay). Method/Kit refers to the published detection method or commercial kit; Technology Platform indicates the detection technology used; Sample Matrix specifies the type of samples tested; Reported LOD denotes the limit of detection as stated by the manufacturer or reported in the published literature.

Method/Kit	Technology Platform	Sample Matrix	Reported LOD
Our method	Double-antibody sandwich ELISA (HRP/TMB colorimetric)	Recombinant protein	3.39 ng/mL
Commercial ELISA Kit	Sandwich ELISA	Serum, plasma, cell culture supernatant	<1.0 ng/mL
Fluorescence immunochromatography method	Time-resolved fluorescence immunochromatography (double-antibody sandwich principle)	Recombinant protein/clinical samples	1.09 ng/mL

## Data Availability

The authors will provide the data related to this manuscript upon request.

## Supplementary Materials

The following supporting information can be downloaded at: https://www.mdpi.com/article/10.3390/diagnostics16132002/s1, Table S1: antibody titer determination Serial dilutions of the antibodies (from 1:100 to 1:204,800 in PBS) were tested in triplicate; Table S2: Determination of antigen concentration in double-antibody sandwich ELISA; Table S3: Determination of optimal enzyme-labeled antibody and coating antibody concentrations; Table S4: Standard curve of the sandwich ELISA; The ARRIVE guidelines 2.0: author checklist.
