# Supplementary material for: Development of a Double-Antibody Sandwich ELISA for the Detection of HPV16 E6 Protein
Source: Diagnostics (Basel). 2026 Jun 26;16(13):2002. doi: 10.3390/diagnostics16132002 (PMC13360185; doi:10.3390/diagnostics16132002)
Supplement: Supplementary file 1 [file diagnostics-16-02002-s001.zip › supplement material.pdf]

Table S1 antibody titer determination Serial dilutions of the antibodies (from 1:100 to 1:204,800 in PBS) were tested in triplicate. The OD_{450} values for each dilution are shown as three independent measurements for A4 and three for G11A11. The titer was defined as the highest dilution giving an OD_{450} value ≥2.1-fold above the negative control.

antibody titer						
dilution	1:100	1:200	1:400	1:800	1:1600	1:3200
	0.5715	0.6226	0.6715	0.622	0.5014	0.4167
G11A11	0.685	0.8039	0.7032	0.5772	0.5082	0.4056
	0.6568	0.7225	0.7255	0.6435	0.5132	0.431
	0.6717	0.6097	0.5141	0.4015	0.2807	0.1955
A4	0.6247	0.6224	0.5201	0.4274	0.3345	0.2262
	0.6721	0.6327	0.5192	0.4128	0.2854	0.2268
NC	0.0599	0.0571	0.0488	0.0559	0.041	0.0581

antibody titer						
dilution	1:6400	1:12800	1:25600	1:51200	1:102400	1:204800
	0.3122	0.2274	0.1505	0.1139	0.1134	0.0982
G11A11	0.3347	0.2424	0.1738	0.1171	0.1285	0.01097
	0.3565	0.31	0.2104	0.1394	0.1016	0.0979
	0.1722	0.1516	0.1487	0.1264	0.11612	0.10431
A4	0.1769	0.1495	0.1497	0.1335	0.1065	0.09425
	0.1578	0.1559	0.1311	0.1269	0.10663	0.09547
NC	0.0557	0.0579	0.0495	0.0407	0.0468	0.0404

Table S2 Determination of antigen concentration in double-antibody sandwich ELISA.OD_{450} values were measured at different concentrations (0, 1, 5, 10μg/mL) of HPV16 E6-His protein to determine the optimal detection concentration.

E6 conc.	0μg/ml	1μg/ml	5μg/ml	10μg/ml
	0.0875	1.6482	2.6986	2.9933
	0.0437	1.6049	2.7432	2.5818
	0.0759	1.3166	2.7332	2.7364

Table S3 Determination of optimal enzyme-labeled antibody and coating antibody concentrations OD_{450} values were measured under various combinations of coating antibody concentrations and HRP-conjugated antibody dilutions to determine the optimal working conditions.

	Coating Ab conc.											
dilution/conc.	1μg/ml			2μg/ml			4μg/ml			8μg/ml		
1/200	1.9178	1.9064	1.8599	2.159	2.2763	2.2481	2.1546	2.1568	1.9618	2.169	2.045	2.3292
1/400	1.6049	1.3796	1.4686	1.9508	1.8361	1.878	2.0296	1.5312	1.6104	2.0614	2.0479	1.8232
1/800	0.9731	1.0089	1.0872	1.3967	1.4266	1.3193	1.2677	1.1111	1.1855	1.3516	1.5117	1.4644
1/1600	0.5455	0.7101	0.6901	0.9921	0.9368	0.8031	1.0522	0.8723	0.8596	0.8284	0.977	0.879
NC	0.0773	0.0625	0.0652	0.0812	0.0716	0.0782	0.0802	0.0703	0.075	0.088	0.0665	0.0849

Table S4 Standard curve of the sandwich ELISA The standard was E6-His protein. PC represents the mean OD value of triplicate measurements for each concentration of the standard (data not shown). NC represents the OD value of PBS blank control. The P/N ratio was calculated as PC divided by NC.

Ag conc.	PC	NC	P/N
187.5	1.220833333	0.03523	34.65323114
93.75	0.873466667	0.03565	24.50116878
46.875	0.507933333	0.03862	13.15208009
23.4375	0.288233333	0.03463	8.32322648
11.71875	0.1749	0.03049	5.736306986
5.859375	0.127066667	0.03483	3.648196009
2.9296875	0.099633333	0.03964	2.513454415
1.46484375	0.0577	0.0343	1.682215743
